# Supplementary material for: Recruitment and Aggregation Capacity of Tea Trees to Rhizosphere Soil Characteristic Bacteria Affects the Quality of Tea Leaves
Source: Plants (Basel). 2024 Jun 18;13(12):1686. doi: 10.3390/plants13121686 (PMC11207862; doi:10.3390/plants13121686)
Supplement: Supplementary file 1 [file plants-13-01686-s001.zip › plants-2993383-supplementary.pdf]

Supplementary data

Table S1 Statistics of splicing results after sequencing of rhizosphere soil bacteria of different varieties of tea trees

Sample ID	Raw tags (Mb)	Clean tags (Mb)	Clean/Raw tags ratio	Sample ID	Raw tags (Mb)	Clean tags (Mb)	Clean/Raw tags ratio
A1	77,304	68,850	89.06%	A23	52,557	47,831	91.01%
A2	275,846	240,991	87.36%	A24	75,958	68,280	89.89%
A3	89,097	80,071	89.87%	A25	86,364	77,311	89.52%
A4	97,592	88,665	90.85%	A26	251,817	217,592	86.41%
A5	126,546	114,810	90.73%	A27	79,913	73,835	92.39%
A6	54,772	50,479	92.16%	A28	168,958	154,237	91.29%
A7	77,943	68,379	87.73%	A29	158,266	148,543	93.86%
A8	142,680	124,637	87.35%	A30	95,667	90,262	94.35%
A9	144,888	127,067	87.70%	A31	130,615	118,540	90.76%
A10	127,508	109,637	85.98%	A32	202,953	180,339	88.86%
A11	79,542	71,447	89.82%	A33	185,646	161,796	87.15%
A12	98,253	85,640	87.16%	A34	209,990	184,682	87.95%
A13	43,911	37,414	85.20%	A35	173,551	161,173	92.87%
A14	178,255	161,276	90.47%	A36	190,293	173,440	91.14%
A15	164,992	147,089	89.15%	A37	152,906	138,333	90.47%
A16	64,504	58,090	90.06%	A38	261,006	241,013	92.34%
A17	47,807	41,711	87.25%	A39	238,871	216,913	90.81%
A18	92,931	84,300	90.71%	A40	99,797	86,630	86.81%
A19	81,543	72,330	88.70%	A41	258,153	251,470	97.41%
A20	171,774	158,846	92.47%	A42	89,557	82,396	92.00%
A21	49,445	44,532	90.06%	A43	279,625	258,526	92.45%
A22	233,523	212,326	90.92%	A44	187,705	170,028	90.58%
Total raw tags					6,150,824 Mb		
Total rlean tags					5,551,757Mb		
Total Clean/Raw tags ratio					90.26%		

Note: Raw_tags are the results of splicing after filtering low-quality fastq data; Clean_tags are the results obtained after removing chimeras and short sequences from the splicing results.

Table S2 Distribution statistics of high-quality sequences after sequencing of rhizosphere soils of different varieties of tea trees

Length(bp) distribute	Sequences reads
0-200	0
200-260	1141
260-320	2115
320-360	3578
360-380	1591
380-400	24127
400-420	4334697
420-440	1181056
440-460	2129
460-480	1073
480-500	118
500-520	93
520-540	39
540-560	0
560-600	0

Table S3 Statistics on the number of OTUs in rhizosphere soils of different varieties of tea trees

Sample ID	Final tags	OTUs	Sample ID	Final tags	OTUs
A1	26546	3068	A23	26546	3513
A2	26546	3774	A24	26546	3710
A3	26546	3555	A25	26546	3535
A4	26546	3589	A26	26546	2829
A5	26546	3325	A27	26546	3007
A6	26546	3772	A28	26546	3340
A7	26546	3832	A29	26546	2655
A8	26546	3486	A30	26546	2924
A9	26546	3318	A31	26546	3398
A10	26546	3560	A32	26546	3862
A11	26546	3301	A33	26546	3961
A12	26546	3237	A34	26546	3293
A13	26546	3450	A35	26546	2427
A14	26546	3466	A36	26546	3324
A15	26546	3483	A37	26546	3321
A16	26546	3482	A38	26546	3634
A17	26546	3553	A39	26546	3350
A18	26546	3482	A40	26546	3545
A19	26546	3198	A41	26546	2593
A20	26546	3532	A42	26546	2913
A21	26546	3627	A43	26546	3272
A22	26546	3517	A44	26546	2752
Total OTUs			147,765		

Note: Final tags is the number of tags corresponding to each sample in the final OTU table; OTUs is the final number of OTUs obtained for each sample.

Table S4 *q*RT-PCR primers for characteristic bacteria

No.	Microorganism	Forward primer	Reverse primer	Product (bp)
1	Candidatus *Udaeobacter*	CTCAATGGGGGAAACCCTGAAG	GACAGGAGTTTACAACCCGAAGA	71
2	*Nitrolancea*	CGCCAGTGGGAAGAGTTCTA	CCGGTACAGTTCACTCCGTG	80
3	*Acidobacteria bacterium*	AACAACCGCCGTTTCCTGAC	TTATCAAGCCGCCTACACGC	133
4	*Chitinophaga*	GTTGGTGAGGTAACGGCTCA	GCCCGTGTGACTGGTCG	70
5	*Rhodoplanes*	GGTACCCACAGAAGAAGCCC	AGTGATTCCGAGCAACGCTA	82
6	*Rhizomicrobium* sp.	GTTGGGAATCTTGGACAATGGG	CTTCATCACTCACGCGGCAT	60
7	*Hyphomicrobiaceae bacterium*	TCAGTTCGGATTGGGCTCTG	TATTCACCGCGCCATGCTG	81
8	*Bryobacter*	ATTCCTGGTGTAGCGGTGAA	GGTCCAGAAAGCCGTCTACA	70
9	*Fimbriimonas*	GGAATCTTGCACAATGGGGG	TACAGGCCTAGACCCTTCGT	70
10	*Haliangium*	AAAGGAATTGACGGGGGC	AACCCAGGTAAGGTTCTGCG	79
11	*Chujaibacter*	AGTCCACGCCCTAAACGATG	ACGCGTTAGCTTCGACACTG	70
12	*Gemmatimonas*	ACAGGTGCTGCATGGCTG	GGTTGCGCTCGTTGCG	80
13	*Acidobacteriaceae bacterium*	TTGTCGGATTTACTGGGCGT	TGAGCCGAGGGATTTCACG	71
14	Candidatus *Koribacter*	TGGGAGAGGTGAGTGGAAT	TTCGCCACAGGTGTTCCTC	71
15	*Acidocella*	AATTCCTGGGCTCAACCTGG	ATTCCACAACCCTCTTCCTCAC	70
16	Candidatus *Solibacter*	GCTCTTTCGGCAGGGAAGAT	TGCTGCCTACGTATTACCGC	89
17	*Rhodospirillaceae bacterium*	AGCCGCGGTAATACGAAGG	GGGAATTTCACGCCTGACTTG	92
18	*Verrucomicrobia bacterium*	TTGTCGTCTGCAACTCGACG	AGGCCTGGGAACGTATTCAC	85

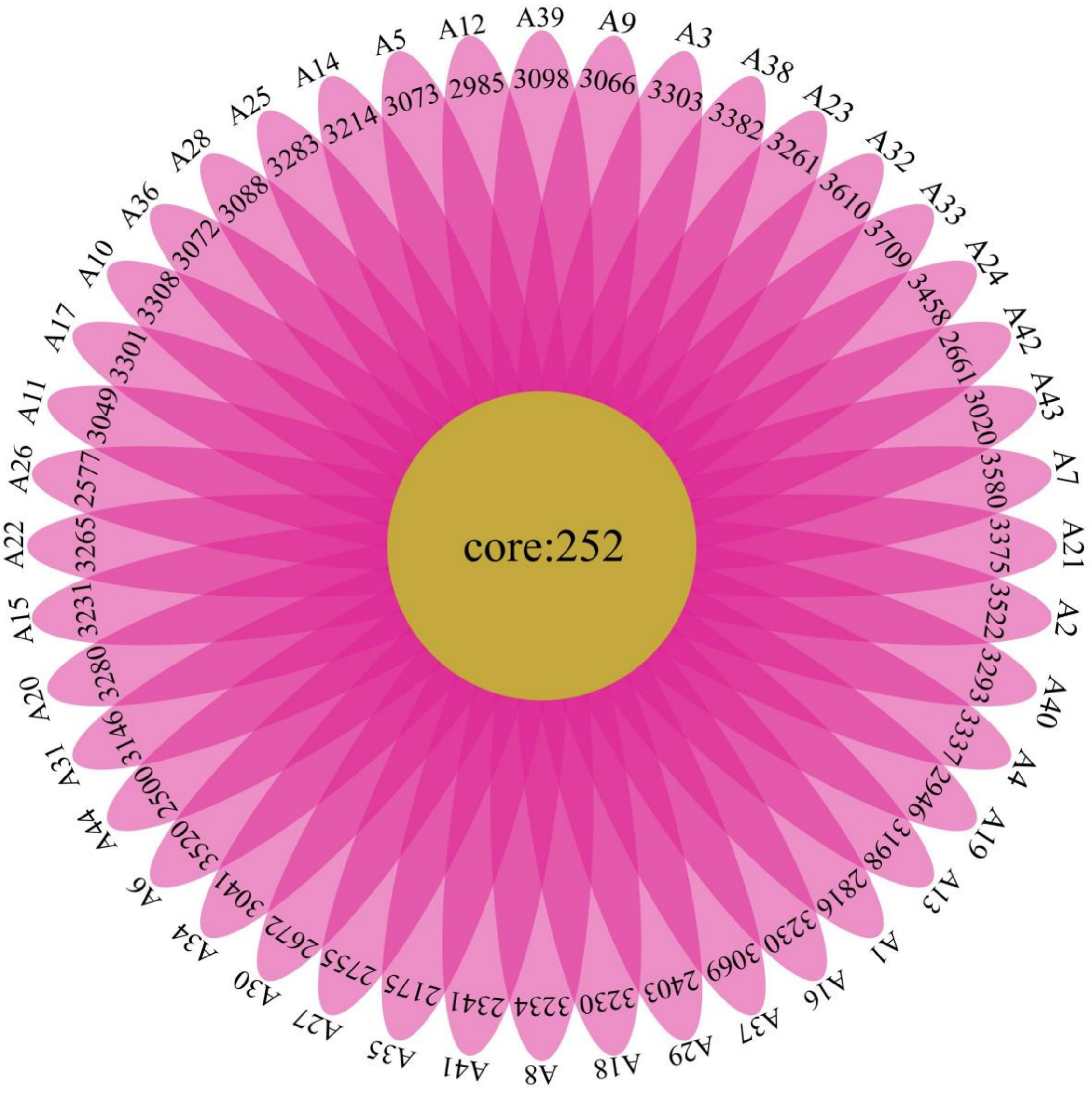

Figure S1. Petalogram analysis of the distribution of rhizosphere soil OTUs of different varieties of tea trees. Each petal represents a tea tree variety, the number in core represents the number of OTUs common to different tea tree varieties, and the numbers on the petals represent the number of OTUs specific to each tea tree variety.

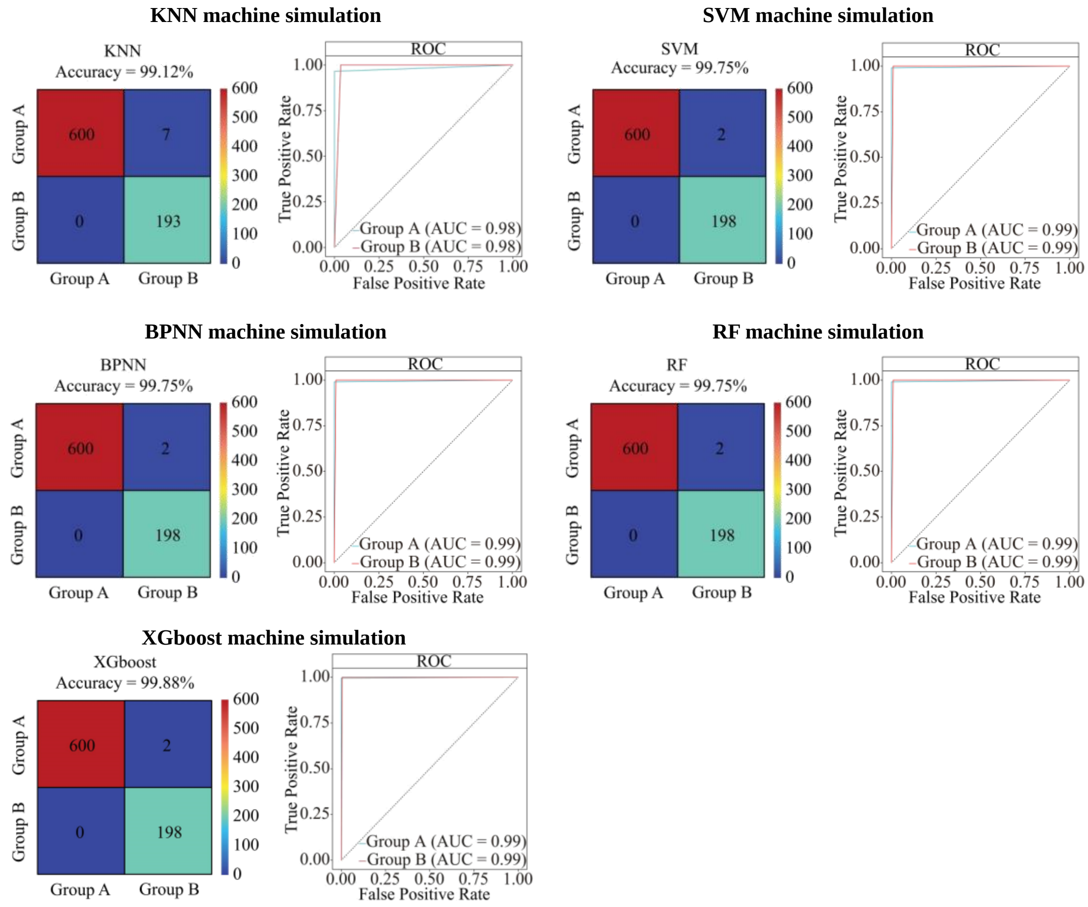

Figure S2. Classification assessment of 44 tea tree germplasm resources based on soil available nutrient content using five machine deep learning methods

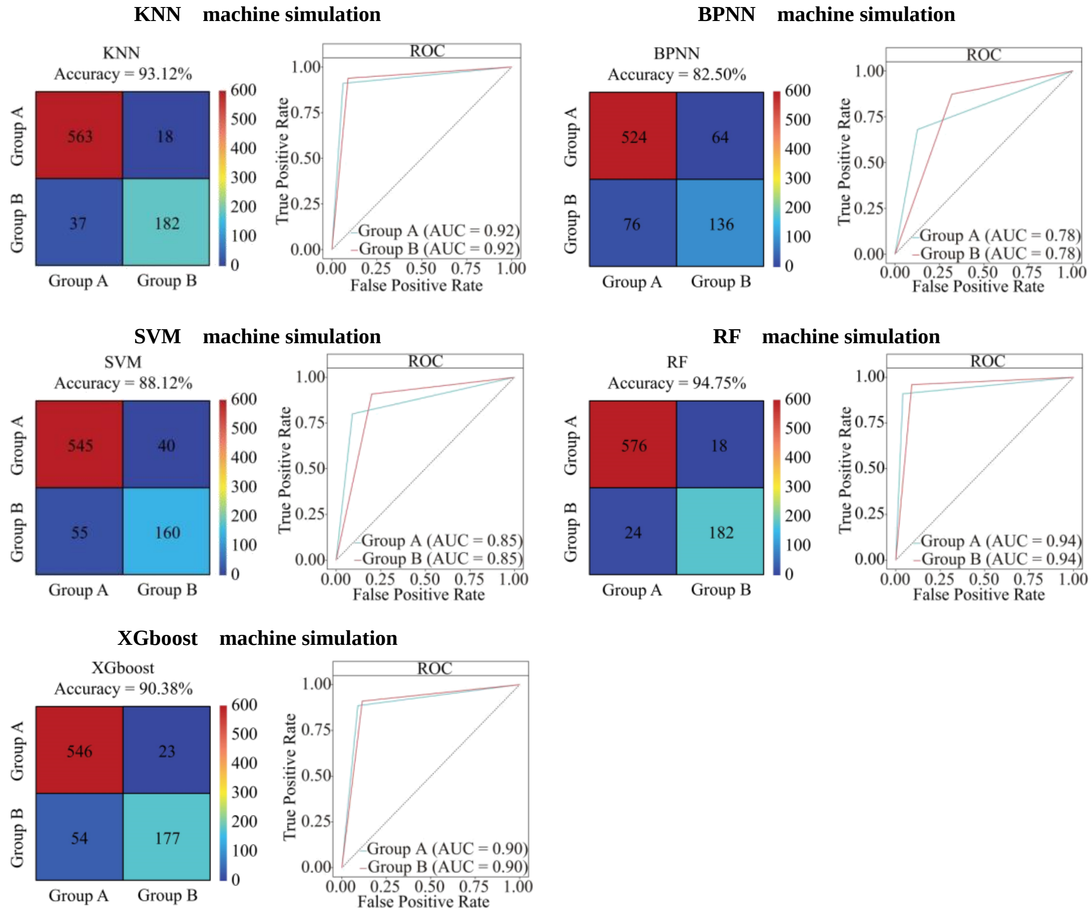

Figure S3. Classification assessment of 44 tea germplasm resources based on tea quality index content using five machine deep learning methods
